# Reproducibility analyses of photo-induced pyroelectric photodetector based on vertically grown SnS layers

**DOI:** 10.1016/j.dib.2018.03.092

**Published:** 2018-03-26

**Authors:** Dong-Kyun Ban, Mohit Kumar, Malkeshkumar Patel, Joondong Kim

**Affiliations:** a Department of Electrical Engineering, Incheon National University, 119 Academy Rd. Yeonsu, Incheon 406772, Republic of Korea; b Photoelectric and Energy Device Application Lab (PEDAL), Multidisciplinary Core Institute for Future Energies (MCIFE), Incheon National University, 119 Academy Rd. Yeonsu, Incheon 406772, Republic of Korea

## Abstract

The data presented in this article includes the photograph of prepared samples and transient photoresponses for 365 and 850 nm wavelengths at different intensities. The original photographs of the working device made of vertically grown SnS layers on Si substrate are presented from the previous results (Kumar et al., 2017, 2018) [1], [2]. Reproducibility measure of the device were checked for thousands of cycles and presented with estimated parameters such as photo current density and photo+pyro current density. Data after analysis are summarized in the table, to profile the photo and pyro responses quantitatively.

**Specifications Table**

**Table:** 

Subject area	Physics, Electrical Engineering
More specific subject area	Solar cells, Photodetector
Type of data	Figures, Table
How data was acquired	Digital camera
	Potentiostat/Galvanostat (ZIVESP1, WonATech,Korea)
Data format	Analyzed
Experimental factors	J-time: Chronoamperometry technique, Self-biased
	Light source:365 and 850 nm, A function generator (MFG-3013A, MCH Instruments) was applied to the light source, Light intensity was calibrated using a power meter (KUSAM-MECO, KM-SPM-11).
Experimental features	Pyroelectric-based SnS/Si photodetector
Data source location	Incheon National University, Incheon-406772, Korea
Data accessibility	The data are with this article

**Value of the data**

1. Photograph of the prepared ITO/SnS/Si/Al devices for the pyroelectric feature and reproducibility of the fabrication.
2. Enhancement in the photocurrent of SnS/Si device for photo-induced pyroelectric effect would be useful to design ultrafast photodetector.
3. Reproducibility and statistical information of the SnS/Si devices would be useful to demonstrate consistency.

## 1 Data

**Fig. 1:**
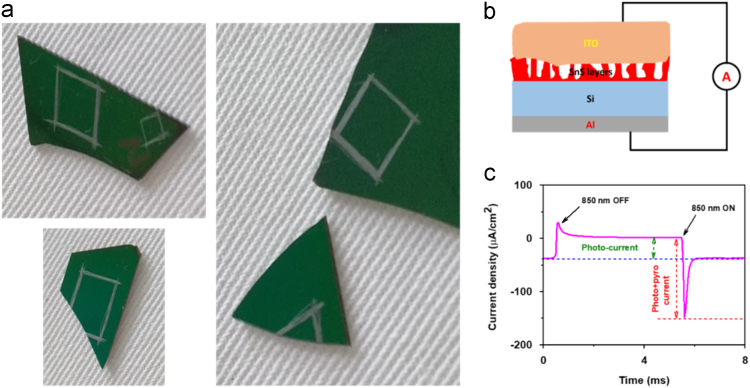
(a) Photographs of the prepared devices to study the reproducibility of vertical SnS layers on Si substrate. (b) Device schematic and (c) Photoresponse of the device at 850 nm.

Fig. 1(a) shows the original photographs of the devices, which was used to study the reproducibility and stability. Fig. 1(b) and (c) are the schematic diagram for of device and measured photoresponse of the device for one cycle, respectively.

**Fig. 2:**
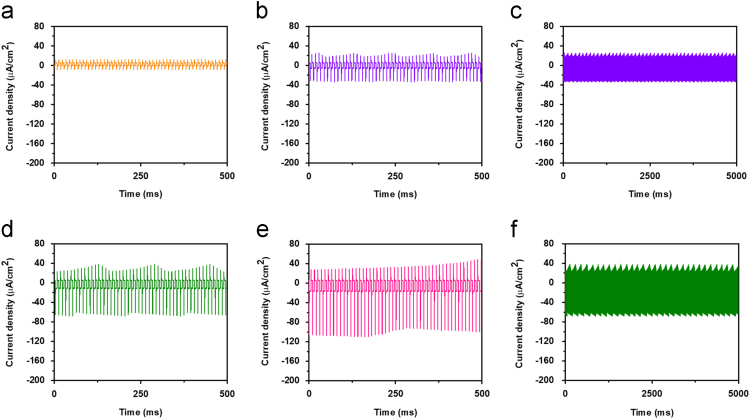
(a), (b), (d) and (e) Depict the photoresponse of the device for 365 nm at different intensities from 1 to 7 mW cm^−2^. (e) and (f) show the transient photoresponse for larger number of cycles of the device for 3 and 5 mW cm^−2^ intensities, respectively.

**Fig. 3:**
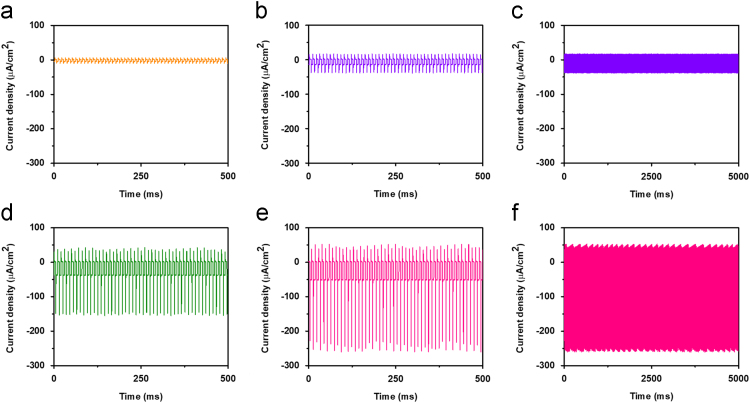
(a), (b), (d) and (e) Depict the photoresponse of the device for 850 nm at different intensities from 1 to 7 mW cm^−2^. (e) and (f) show the transient photoresponse for larger number of cycles of the device for 3 and 7 mW cm^−2^ intensities, respectively.

The presence of peaks during the light ON and OFF condition is attributed to the photon-induced pyroelectric effect [1], [2], [3], [4]. Fig. 2 shows the response of the device at 365 nm at different intensities for large number of cycles, confirming high reproducibility of the same. Fig. 3 depicted the photoresponse of the SnS/Si device for 850 nm at different intensities. Estimated parameters such as photocurrent and photo+pyro current densities for 365 and 850 nm for different intensities are summarized in Table 1.

**Table 1:** Intensity dependent photocurrent (*J*_Ph_) and photo+pyro current (*J*_Ph+Py_) densities for 365 and 850 nm.

Intensity (mW cm^−2^)	365 nm		850 nm	
	*J*_Ph_ (µA)	*J*_Ph+Py_ (µA)	*J*_Ph_ (µA)	*J*_Ph+Py_ (µA)
1	6	18	6	12
2	10	36	14	38
5	15	71	37	154
7	20	114	51	257

## 2 Experimental design, materials and methods

### 2.1 Sample preparation

*n*-type Si substrates were used as substrates to prepare the device and was cleaned according to Refs. [1,2]. Vertical SnS layers were formed using the RF magnetron sputtering. Conditions for preparing SnS sample is as follows.

**Table:** 

Target	SnS_2_ target (iTASCO, TSNALT0027, ∅ 2-inch)
RF power	50 W
Gas/flow rate	50 sccm
Deposition pressure	6 mTorr
Temperature	300 °C
Substrate rotation	5 rpm

### 2.2 Sample characterizations

Two different light sources of ultraviolet (365 nm) and near-infrared (850 nm) were used in the photoresponse measurements. The top ITO layer and back Al contact were connected to the positive and the negative terminals of the SMU, respectively. The transient photoresponse of the device was studied by the chronoamperometry method under pulsed monochromatic light by varying the bias and light intensity. A function generator (MFG-3013A, MCH Instruments) was applied to the light source. Light intensity was calibrated using a power meter (KUSAM-MECO, KM-SPM-11). The current-voltage characteristics and responsivity were also confirmed by performing the measurements using Keithley 2440 source meter.

## References

[1] Kumar M., Patel M., Kim J., Kim J., Kim B. (2018). Vertically aligned crystalline SnS layers-based NIR photodetector governed by pyro-phototronic effect. Mater. Lett. Mater. Lett..

[2] Kumar M., Patel M., Kim J., Lim D. (2017). Enhanced broadband photoresponse of a self-powered photodetector based on vertically grown SnS layers via the pyro-phototronic effect. Nanoscale.

[3] Wang Z., Yu R., Pan C., Li Z., Yang J., Yi F., Wang Z.L. (2015). Light-induced pyroelectric effect as an effective approach for ultrafast ultraviolet nanosensing. Nat. Commun..

[4] Kumar M., Patel M., Lee G.N., Kim J. (2018). Light-induced all-transparent pyroelectric photodetector. ACS Appl. Nano Mater..

