# Supplementary material for: Reproducibility analyses of photo-induced pyroelectric photodetector based on vertically grown SnS layers
Source: Data Brief. 2018 Mar 26;18:790–4. doi: 10.1016/j.dib.2018.03.092 (PMC5996739; doi:10.1016/j.dib.2018.03.092)
Supplement: Supplementary file 1 — Supplementary material [file mmc1.docx]

January 31, 2018

Managing Editor

Data in Brief

Dear Editor

DIB-D-17-01146

Title: Reproducibility analyses of photo-induced pyroelectric photodetector based on vertically grown SnS layers

Authors: Dong-Kyun Ban, Mohit Kumar, Malkeshkumar Patel, and Joondong Kim

We declare that this manuscript is original, has not been reported before, and is not currently being considered elsewhere. We also confirm that there is no known conflict of interest regarding this manuscript and its publication. The manuscript has been approved by all named authors.

Sincerely yours,

Joondong Kim

--------------------------------------------------------------------

Ph. D./Professor

Department of Electrical Engineering

Incheon National University

119 Academy Rd. Yeonsu Incheon, 406772, Korea

Email: [joonkim@inchoen.ac.kr](mailto:joonkim@inchoen.ac.kr) Tel: +82-32-835-8770
